# “Thanks for hearing me out”: Voices of social work students during COVID-19

**DOI:** 10.1177/1473325020981073

**Published:** 2021-03

**Authors:** Sarah J Cole, Samantha S Mitra, Jennifer A Robinson, Sarah Jen, Megan S Paceley, Kortney A Carr, Michael Riquino, Kelechi Wright

**Affiliations:** School of Social Welfare, University of Kansas, Lawrence, USA

**Keywords:** Research poem, pandemic, social work students, social work education, COVID-19

## Abstract

As social work educators and students, the COVID-19 pandemic impacted our teaching and learning in challenging ways. We embarked on a qualitative research study to better understand the ways in which the pandemic was affecting the social work students in our program. Three faculty mentors worked collaboratively with five social work students across BSW, MSW, and PhD programs to interview 66 BSW and MSW students about their experiences, challenges, and hopes during the early months of the pandemic. BSW and MSW students led the analysis and early dissemination for the project. This essay describes the unique experiences of social work students by using a research poem to capture the emotional and experiential aspects of the students we interviewed.

As social work students, the COVID-19 pandemic impacted our personal, educational, and collective experiences on a fundamental level. Not only did we feel individual distress, but we were also deeply cognizant of the suffering of others due to this pandemic. We struggled to make meaning out of our experiences during this time. And so, when we were approached by two social work professors who wanted to understand the experiences of social work students during COVID-19, we welcomed the opportunity to participate in the research process. Within an hour of sending out an email call for participants, we had nearly 100 students request an interview. We felt this demonstrated a desire to process their pandemic-related experiences, which were often isolating and disorienting. Our faculty mentors wanted to center the voices of students not only in the data but also in the process, and so we were offered the opportunity to take on leadership of the analysis of the 66 individual narratives collected. Throughout the process, we found ourselves reflecting upon our own experiences as students during this pandemic, allowing them to guide us in identifying themes potentially unique to social work students. We identified a collective, unifying story that we felt needed to be heard. We chose to share that story in a research poem because it not only centered the voices of the participants, but also allowed us to highlight the emotional quality and lyrical nature of our participants’ reflections. In the depth of experience that was shared with us, we saw glimmers of our own lives like points of commonality that connected us across isolation, collective trauma, and a desire to work towards mutual well-being.

I pursued social work because I have a deep passion

for social justice…I got tired of being angry

all the time and not actually doing anything

about it…There are some difficulties I experienced in life

that led me to have a traumatic brain injury

I had a social worker advocate for me

Right there, in that moment, I decided

I wanted to do that. I wanted to be that.

Before the pandemic, I felt like I had a voice in class

I was enriched by the conversations…I’d get up, go to work

go to night class, straight from work to class

change in my car, eat in my car. I’d get home, shower,

go to bed, wake up, do the same thing.

It was constant. I felt like I was never home.

I don't really feel like I had a transition, I think it was a shift

in reality…I feel like we all, at the beginning, felt like

this wasn't going to touch us…March was this month

of piecemeal information…How does this transmit?

How do we protect ourselves?

What does it mean?

In my young adult life, I have never *not* wanted to be an adult more. I was like, “I want somebody else to figure this out for me.”

Somebody leaked that the school was going to give us another

two weeks for, kind of the way they spun it was, spring break.

And I was like, “I have enough common sense to realize

this isn't a get out of jail free card.” This is really serious.

All my anchors are gone. I mean, that's how I described it.

I got zero termination with my kids, and I feel

like that's such an unethical thing to do, to terminate

without any type of… anything?

My client actually passed away. I think that's something that’s been hard

is no funerals…It was hard to not be a part of that celebration of life.

I feel a shrinkage of my life.

I keep seeing motivational statements, well thought-out,

articulate statements about how we’re going to save the world

as long as we stick together.

I’m just not feeling that right now.

It’s sad to feel a little bit forgotten sometimes, they forget

the rural population doesn’t have resources or hospitals

close… I’m also immunocompromised, so the stress of going

to the store or just being around people went to a thousand

times the typical stress level that I would usually have.

Fearing for my own safety at the grocery store with 500 other people

Am I going to be part of a documentary some day?

“All of these people didn’t know they were going to die going to the store.”

Anxiety exacerbated.

You can try coping skills and things you’ve learned in class, but it’s not the same. I guess I can’t be my own therapist.

If you don’t let me go outside

at least walk in our neighborhood, I will lose

my mental state…We need air. We need to see the sky.

I am safer over here

in my house by myself,

but if I get sick and never get to see you again…

How do you balance that?

Even with my closest relationships, I felt that my

trust was shaken…It completely throws

your identity upside down.

It's like a social death.

I think I've bonded with my classmates incredibly over

this though. I think we all just, we had the best time

poking fun at our situation. All of us said

we went to Zoom University.

The extra work that my brain was doing to try to read people

on a screen, at the end of the day,

I'd be like, “Oh my God, I can't look at another screen.”

It just felt trivial

to do these assignments…

…when everything around you is chaos.

I feel the crushing weight of the collectivism

of our culture…how it doesn't matter if one of us

is doing well, if the others are suffering.

We all have to do well together.

As a social worker, I feel that innate need to do something

about it, but there's nothing I can do

other than keeping myself safe.

And then I had guilt

because of having health issues…I didn't know what I was going

to be able to do, I was feeling bad

about not being able to be involved.

There was this initial push for everyone

to be like, “We’ve got to return back to normal

as soon as possible.” Then one day it dawned on me

that we didn't, we could rebuild the system.

It’s important for people to recognize that everybody’s essential in some aspect.

This isn't ending.

I mean, whatever happens

after this, we're ricocheting

off of this moment.

*Thanks for hearing me out.*

